# Camel Milk Resistome in Kuwait: Genotypic and Phenotypic Characterization

**DOI:** 10.3390/antibiotics13050380

**Published:** 2024-04-23

**Authors:** Rita Rahmeh, Abrar Akbar, Batlah Almutairi, Mohamed Kishk, Naida Babic Jordamovic, Abdulaziz Al-Ateeqi, Anisha Shajan, Heba Al-Sherif, Alfonso Esposito, Sabah Al-Momin, Silvano Piazza

**Affiliations:** 1 Environment & Life Sciences Research Center, Kuwait Institute for Scientific Research, P.O. Box 24885, Safat 13109, Kuwait; aajakbar@kisr.edu.kw (A.A.); bmutairi@kisr.edu.kw (B.A.); mwaheed@kisr.edu.kw (M.K.); aateeqi@kisr.edu.kw (A.A.-A.); ashajan@kisr.edu.kw (A.S.); hshareef@kisr.edu.kw (H.A.-S.); smomen@kisr.edu.kw (S.A.-M.); 2 Computational Biology, International Centre for Genetic Engineering and Biotechnology, 34149 Trieste, Italy; naida.jordamovic@icgeb.org (N.B.J.); silvano.piazza@icgeb.org (S.P.); 3 Faculty of Agricultural Sciences, Free University of Bozen-Bolzano, 39100 Bolzano, Italy; alfonso.esposito@icgeb.org

**Keywords:** shotgun sequencing, resistome, antibiotic resistance genes, mobile genetic elements, camel milk

## Abstract

Antimicrobial resistance (AMR) is one of the major global health and economic threats. There is growing concern about the emergence of AMR in food and the possibility of transmission of microorganisms possessing antibiotic resistance genes (ARGs) to the human gut microbiome. Shotgun sequencing and in vitro antimicrobial susceptibility testing were used in this study to provide a detailed characterization of the antibiotic resistance profile of bacteria and their ARGs in dromedary camel milk. Eight pooled camel milk samples, representative of multiple camels distributed in the Kuwait desert, were collected from retail stores and analyzed. The genotypic analysis showed the presence of ARGs that mediate resistance to 18 classes of antibiotics in camel milk, with the highest resistance to fluoroquinolones (12.48%) and disinfecting agents and antiseptics (9%). Furthermore, the results pointed out the possible transmission of the ARGs to other bacteria through mobile genetic elements. The in vitro antimicrobial susceptibility testing indicated that 80% of the isolates were resistant to different classes of antibiotics, with the highest resistance observed against three antibiotic classes: penicillin, tetracyclines, and carbapenems. Multidrug-resistant pathogens including *Klebsiella pneumoniae*, *Escherichia coli*, and *Enterobacter hormaechei* were also revealed. These findings emphasize the human health risks related to the handling and consumption of raw camel milk and highlight the necessity of improving the hygienic practices of farms and retail stores to control the prevalence of ARGs and their transmission.

## 1. Introduction

Antimicrobial resistance (AMR) is a growing global threat that has significant impacts on human health and the economy [1]. It is a major public health challenge in the 21st century. If not tackled and effective strategies are not implemented, it is estimated that AMR will cause 10 million deaths per year with a cumulative cost to the world economy of 100 trillion USD by 2025 [2]. This challenge no longer solely concerns human health; rather, the emerging AMR concern necessitates a “One Health” strategy that considers human, animal, and environmental reservoirs [3]. Many infectious microorganisms acquire resistance to most antibiotic classes to which they were previously sensitive [4]. These microorganisms evolve over time, develop new resistance mechanisms, and cease responding to antibiotics. This makes infections difficult to treat and increases the risk of disease transmission and death in animals and humans [5]. AMR prevalence is globally increasing owing to the widespread overuse of antibiotics in human healthcare, animal growth promotion, and veterinary disease control [2]. An overwhelming spread of multidrug-resistant (MDR) strains is likely to emerge as a result of these tendencies.

Food contamination by pathogens, along with the consideration of food-producing animals as reservoirs of pathogenic bacteria, including MDR bacteria, cause significant health issues and economic losses worldwide [6]. Over the past few years, there has been a significant increase in the occurrence of MDR pathogens in foods of animal origin, including milk, meat, and poultry [7]. These MDR microorganisms may be transmitted to humans during the processing or consumption of raw animal products. Consuming unprocessed animal products such as raw milk increases the possibility of microorganisms possessing transferable antibiotic resistance genes (ARGs) being transmitted to the human gut microbiome [8]. ARGs may be shared by horizontal gene transfer of mobile genetic elements among animals, humans, and the environment [2]. Raw milk contamination with zoonotic pathogens including MDR microbes has been well documented as leading to serious diseases [2,8,9]. Despite the potential risk of infection associated with drinking raw milk, camel milk is consumed raw because of its known therapeutic properties against many diseases and the long-standing belief that heat treatment reduces its nutritional and medicinal value [10]. Despite the health benefits of raw camel milk for humans, bacteria causing spoilage and/or illnesses have been identified in raw camel milk using 16S rRNA gene amplicon sequencing [11]. A previous study reported dominant genera that may include species belonging to ESKAPE organisms, including *Enterococcus faecium*, *Staphylococcus aureus*, *Klebsiella pneumoniae*, *Acinetobacter baumannii*, *Pseudomonas aeruginosa*, and *Enterobacter* spp., which exhibit multidrug resistance and virulence [12]. AMR in ESKAPE organisms is usually associated with high mortality and economic costs. Directing attention toward these organisms can help address the broader threat of AMR [13]. Furthermore, a recent study demonstrated that Bactrian raw camel milk serves as a reservoir for ARGs using high-throughput quantitative PCR and 16S rRNA gene-based Illumina sequencing [14]. Consequently, the consumption of raw camel milk might promote the spread of MDR bacteria and ARGs to the food chain and humans. If preventive measures and efficient solutions are not nationally applied, the worldwide spread of AMR will endanger AMR policies in other parts of the world. Thus, there is an urgent need to understand the national AMR status to efficiently control the proliferation of MDR bacteria.

The microbiome and resistance pattern of milk can be evaluated using culture-dependent and culture-independent methods. Culture-based resistance determination is based on the isolation of target bacteria and the evaluation of the growth in the presence of antibiotics. However, this method only reveals a subset of species present in the sample, as the growth of a large number of bacteria is difficult or even impossible under standard conditions [15]. As a consequence, the culture-dependent method underestimates species and, therefore, the AMR genes. Recent advances in high-throughput sequencing and bioinformatics analysis have offered this field an advantage by allowing rapid AMR gene identification and characterization while overcoming the bias of the culture-dependent method [16]. Shotgun metagenomic sequencing involves unrestricted sequencing of the genomes of all microorganisms within a sample, including uncultured ones, resulting in a significant amount of data that can be analyzed to determine whether AMR genes are present [17]. This technology successfully gives insight into the bacterial composition and functional diversity in a sample. The presence of AMR genes revealed by shotgun sequencing can be used to predict the phenotypic resistance of microorganisms; however, potential inconsistency may arise when performing culture-based resistance determination [18]. Although the majority of metagenomics approaches have been devoted to short-read sequencing, interest in the new opportunities provided by third-generation sequencing technologies, which generate longer sequencing reads, is growing. Thus, a comprehensive understanding of the resistance challenge requires a combination of culture-based and shotgun sequencing approaches, as these two methods measure different parameters, with shotgun sequencing typically focusing on the characterization of the entire microbial community, whereas the culture-based approach tests the response of individual isolates to specific antibiotic doses [19].

At present, little is known about antibiotic-resistant bacteria and their resistance genes in raw dromedary camel milk. In this study, the genotypic and phenotypic resistance in raw camel milk from retail stores has been examined using complementary shotgun sequencing and culture-based techniques. The ARG profiles of raw camel milk samples collected from retail stores in Kuwait, their mechanism of action, and their potential transmission among bacteria were characterized using shotgun sequencing technology. Moreover, antimicrobial susceptibility testing was further employed to determine the phenotypic resistance profile of the bacteria isolated from camel milk.

## 2. Results

### 2.1. Genotypic Resistance Profile of Camel Milk

In order to reduce the AMR prevalence, a comprehensive understanding of the antimicrobial resistance in food that has a significant role in the cross-border and sectoral global spread of antimicrobial resistance is essential. Raw camel milk’s contribution to the spread of AMR is unknown. This knowledge gap needs to be addressed. The shotgun sequencing technique enables the study of the resistome of multiple foods, including dairy products [3,20]. Herein, we employed a complementary culture-based and metagenomics approach to study the resistant bacteria and their AMR genes in raw camel milk samples collected from retail stores and evaluate the potential transmission of these ARGs.

The presence and relative abundance of AMR genes were studied using shotgun sequencing that yielded 0.4 billion raw reads, with an average of 48.5 million raw reads per sample (range 48.3–48.7 million). The percentage of host reads was 44.4% (Supplementary Table S1).

**Figure 1:**
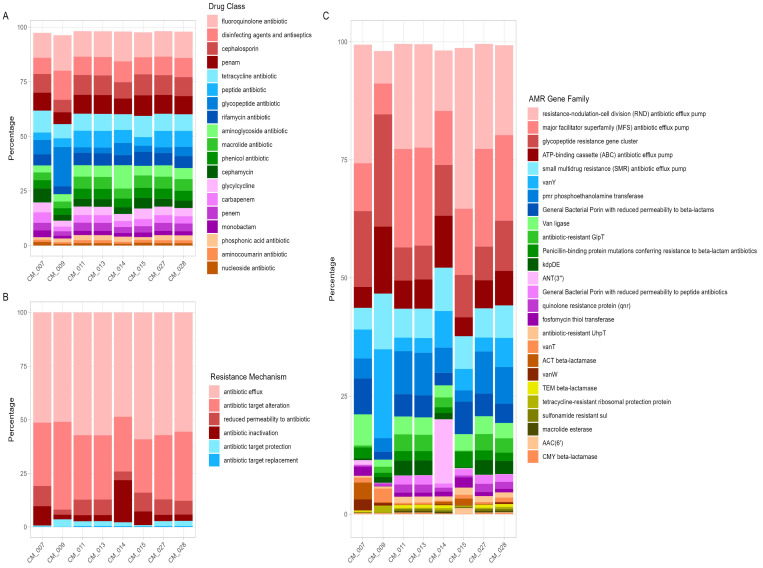
Antibiotic resistance profile determined by shotgun sequencing. Relative abundances of drug classes (**A**), resistance mechanisms (**B**), and AMR gene families (**C**) across samples.

**Figure 2:**
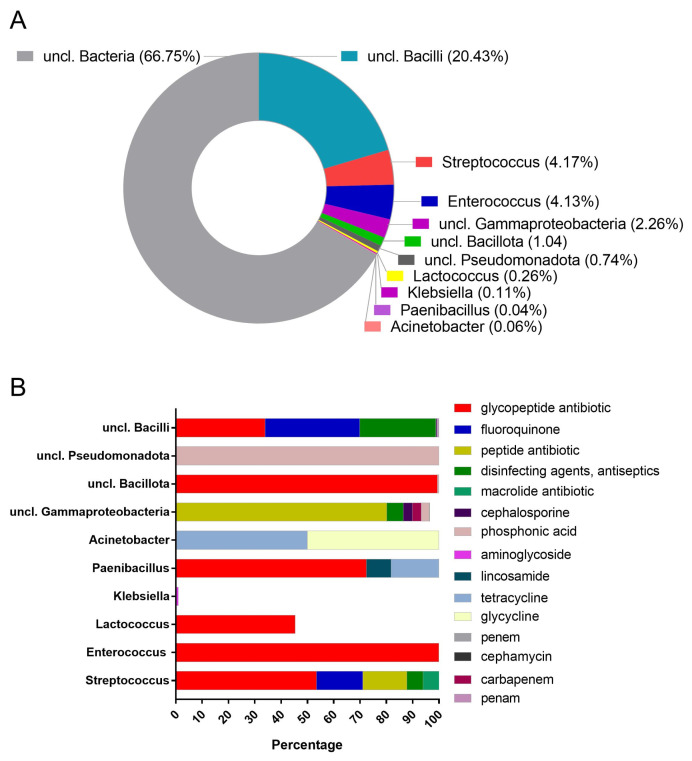
Percentage of bacterial genus relative abundance (**A**), and percentage of drug class relative abundance within each genus (**B**).

The resistance genes were predicted using the CARD Resistance Gene Identifier [21]. In total, 18 AMR classes and 26 AMR gene families with a group mean relative abundance of more than 1% and 0.2%, respectively, were identified across all analyzed samples (Figure 1 and Figure 2). The most frequently detected AMR classes across the majority of the samples were fluoroquinolones (12.48%), cephalosporins (8.42%), penam (8%), tetracycline (8%), peptide antibiotics (5.92%), glycopeptide antibiotics (5.62%), rifamycin (5.29%), and aminoglycoside (5.27%). Genes of resistance for the drug classes macrolide (4.84%), phenicol (4.25%), cephamycin (3.87%), glycylcycline (3.33%), carbapenem (3.35%), and penem (3.21%) were the next most prominent group. The third most abundant detected genes of resistance were against monobactam (2%), phosphonic acid (1.92%), aminocoumarin (1.18%), and nucleoside antibiotics (1.13%). Resistance genes against additional antibiotic classes with a mean relative abundance of less than 1% are presented in Table 1. Moreover, genes resistant to disinfecting agents and antiseptics (9%) were also detected among the most dominant resistant genes (Figure 1A).

The resistance mechanism profiles depicted in Figure 1B show that antibiotic efflux (54.62%) and antibiotic target alteration (30.4%) were the major AMR mechanisms, in addition to other mechanisms including reduced permeability to antibiotic (6.57%), antibiotic inactivation (6.13%), antibiotic target protection (1.9), and antibiotic target replacement (0.37%).

The bacterial hosts of ARGs detected on de-novo-assembled contigs were identified using the Contig Annotation Tool (CAT) v5.2.3 [22] based on database matches inferred by DIAMOND during gene annotation. Taxonomic information was taken from the pre-compiled CAT taxonomy database of 2021-01-07. At the genus level, 10 bacterial genera were predicted to host ARGs, namely unclassified *Bacilli* (20.43%), *Streptococcus* (4.17%), *Enterococcus* (4.13%), *unclassified Gammaproteobacteria* (2.26%), *unclassified Bacillota* (1.04%), *unclassified Pseudomonadota* (0.74%), *Lactococcus* (0.26%), *Klebsiella* (0.11%), *Acinetobacter* (0.06%), and *Paenibacillus* (0.04%) (Figure 2A). The percentage of drug class relative abundance within each genus from which the identified resistance determinants originated is represented in Figure 2B.

Taxonomic assignment of contigs revealed the presence across all samples of different AMR genes conferring resistance to glycopeptides (the vanY gene in the vanB cluster, and the vanT gene in the vanG cluster) through an antibiotic target alteration mechanism. These AMR genes have been taxonomically assigned at the genus level to *Enterococcus*. Genes mediating resistance to glycopeptide (the vanW gene in the vanG cluster, the vanY gene in the vanF cluster, and the vanY gene in the vanM cluster) and peptide antibiotic (*Streptococcus agalactiae* mprF) through antibiotic target alteration were identified. Resistance to macrolide (mreA) and fluoroquinolone (patB) in addition to multidrug resistance to disinfecting agents and antiseptics (qacJ) via antibiotic efflux was also revealed. These AMR genes were linked to the *Streptococcus* genus. The AMR gene adeC, conferring resistance to glycylcycline and tetracycline via antibiotic efflux, was detected in *Acinetobacter*. AMR genes with resistance to lincosamide (lmrD) and glycopeptide (the vanY gene in the vanG cluster) mediated by antibiotic efflux and antibiotic target alteration, respectively, were associated with *Lactococcus.* In sample CM_014, genes of resistance to glycopeptide (the vanY gene in the vanB cluster, and the vanW gene in the vanI cluster) and tetracycline (tet(36)) through antibiotic target alteration and antibiotic target protection, respectively, were found to be associated with *Paenibacillus*. In sample CM_015, multidrug resistance genes to aminoglycoside (CrcB) belonging to the multidrug and toxic compound extrusion (MATE) transporter gene family that excreted resistance through antibiotic efflux were linked to *Klebsiella* (Table 2).

To investigate the transferability of AMR genes, the Platon tool was used to identify the resistance genes located on the plasmid [23]. Several genes of resistance for lincosamide (lnuB); lincosamide, streptogramin, and pleuromutilin (lsaE, lsaE); tetracycline (tet(A), tet(45)), macrolide (EreA); disinfecting agents and antiseptics (qacEdelta1, qacL); sulfonamide (sul1, sul3); monobactam, cephalosporin, penam, and penem (TEM-1); cephalosporin and penam (CTX-M-15); cephalosporin and cephamycin (DHA-1); cephalosporin, penam, and penem (LAP-2); phenicol (cmlA1); fluoroquinolone (QnrS1); fluoroquinolone and aminoglycoside (AAC(6′)-Ib-cr6); aminoglycoside (APH(3′)-IIIa); diaminopyrimidine (dfrA12); and macrolide, lincosamide, streptogramin, streptogramin A, and streptogramin B (ErmB) were predicted to be carried on plasmids. These resistance genes were surrounded by mobile genetic elements, and some of these genes are part of insertion sequences and transposons, such as ISRe46, Tn2, ISArsp14, MICBce1, ISAba61, ISBce8, ISCco2, and ISSsu9, as identified by ISFinder. For example, the resistance gene lsaE conferring resistance to lincosamide, streptogramin, and pleuromutilin antibiotics is part of the insertion sequence ISRe46 and is surrounded by two other mobile elements: one positioned as the 6th CDS downstream and the other as the 2nd CDS upstream. ISRe46 belongs to the IS481 family, which occurs widely in bacteria and may be linked to regulators and determinants of antibiotic resistance, resulting in self-mobilizable transposons [24]. The list of these resistance genes, their resistance mechanism of resistance to drug classes, and their distance to mobile genetic elements are presented in Table 3.

Additionally, resistance genes within a 10-CDS distance of mobile genetic elements were associated with bacterial genera. The AMR genes, including the vanT gene in the vanG cluster, the vanW gene in the vanI cluster, tet(36), the vanY gene in the vanB cluster, LlmA 23S ribosomal RNA methyltransferase, tet(45), and the vanH gene in vanO cluster, were associated with *Paenibacillus.* The vanY gene in the vanB cluster and the vanT gene in the vanG cluster were linked to *Enterococcus*. The vanY gene in the vanM cluster, patB, *Streptococcus agalactiae* mprF, mreA, and qacJ were related to *Streptococcus*, and lmrD to *Lactococcus* (Table 4).

### 2.2. Phenotypic Resistance Profile of Camel Milk

A total of 33 isolates were obtained from the samples and genetically identified as *Klebsiella pneumoniae* (*n* = 12) (36%), *Escherichia coli* (*n* = 5) (15%), *Acinetobacter* sp. (*n* = 8) (24%), *Enterobacter* sp. (*n* = 5) (15%), *Citrobacter* sp. (*n* = 1) (3%), and *Enterococcus* sp. (*n* = 2) (6%). Notably, species that are commonly detected in raw milk such as *Listeria monocytogenes*, *Staphylococcus aureus*, *Streptococcus* sp., *Salmonella* sp., and *Pseudomonas* sp. were not identified in these samples.

**Figure 3:**
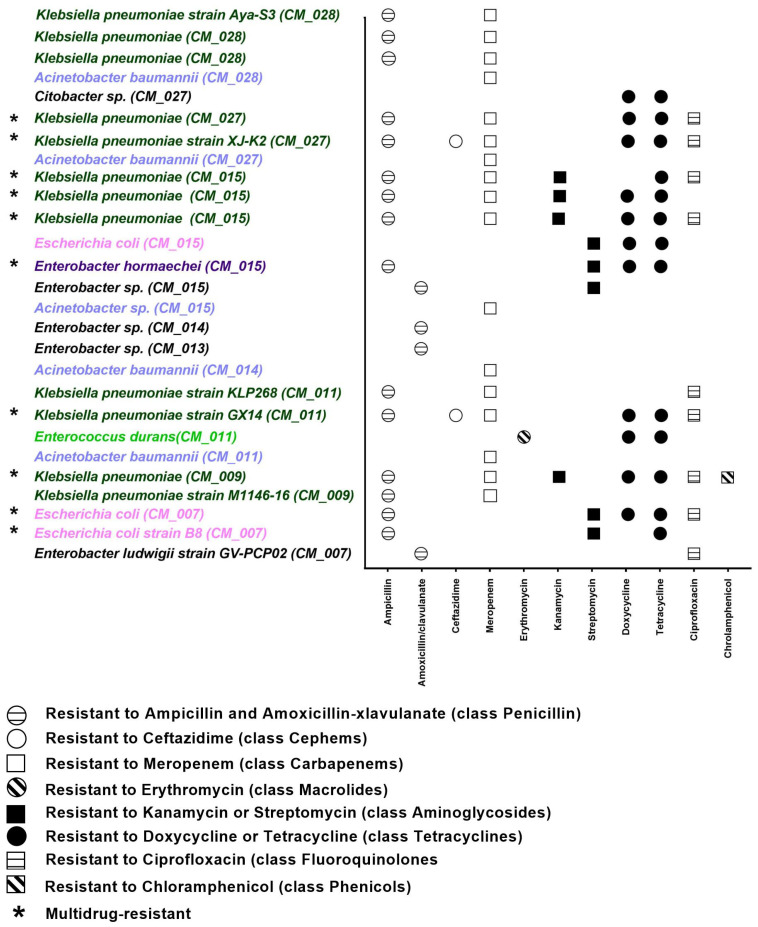
Antibiotic resistance profile of 27 isolates from camel milk against 8 antibiotic classes determined using the disk diffusion method.

The antimicrobial resistance of these isolates was tested using the disc diffusion method, and the results were interpreted according to the CLSI guidelines [25]. Among these isolates, 27 resistant strains were identified including 10 MDR isolates. The resistant strains were identified as *Klebsiella pneumoniae* (*n* = 12), *Escherichia coli* (*n* = 3), *Acinetobacter baumannii* (*n* = 4), *Acinetobacter* sp. (*n* = 1), *Enterobacter* sp. (*n* = 4), *Enterobacter hormaechei* (*n* = 1), *Citrobacter* sp. (*n* = 1), and *Enterococcus durans* (*n* = 1). The antibiogram profiling revealed that 10 of the 33 isolates (33%) were MDR, exhibiting resistance to at least three classes of antibiotics [26]. The MDR isolates were *Klebsiella pneumoniae* (*n* = 7), *Escherichia coli* (*n* = 2), and *Enterobacter hormaechei* (*n* = 1). The isolates of *Klebsiella pneumoniae* demonstrated the highest resistance to ampicillin and meropenem (100%) and moderate resistance to the tetracycline class (tetracycline (58%) and doxycycline (50%)). These isolates also showed resistance to ceftazidime, kanamycin, ciprofloxacin, and chloramphenicol, ranging from 8 to 33%. The *E. coli* isolates showed moderate resistance to streptomycin, tetracycline, doxycycline, and ampicillin (40–60%) and resistance to ciprofloxacin (20%). Moreover, the isolates of *Enterobacter* sp. exhibited high resistance to amoxicillin/clavulanate (80%) and moderate resistance to streptomycin (40%) in addition to their resistance to tetracycline, doxycycline, ciprofloxacin, and ampicillin ranging from 20 to 40%. The isolates of *Acinetobacter baumannii* showed the highest resistance against meropenem (100%) while being sensitive to all the other tested antibiotics. The antimicrobial resistance patterns of the isolates against the antibiotics used in this study are represented in Figure 3, and the resistance profiles of the *Enterobacterales* are summarized in Table 5. All the isolates were sensitive to imipenem antibiotic. In this study, the antibiogram profile demonstrated that the highest resistance was against three classes of antibiotics, penicillin, tetracyclines, and carbapenems (Figure 3 and Table 5).

## 3. Discussion

Antimicrobial resistance is a global concern. The emergence of AMR in food and the possibility of ARG-carrying microorganisms invading the human gut microbiome represent a significant risk to human health. Camel milk is consumed raw despite the potential risk of infection owing to its known therapeutic properties against numerous diseases and the consumer belief that heat treatment impairs its nutritional and medicinal properties. The available information on the antibiotic resistance profile of raw dromedary camel milk is limited, and the contribution of this milk to the spread of AMR is unknown. To fill this knowledge gap, a combination of both shotgun sequencing and culture-based techniques was employed in this study to investigate the genotypic and phenotypic resistance in raw camel milk from retail stores in Kuwait. The shotgun sequencing approach is currently being widely used to elucidate the genes encoding resistance to antibiotics in milk [3,20]. Previously, using 16S rRNA gene amplicon sequencing, we reported that raw camel milk contains bacteria that cause spoilage and/or illnesses [11]. These bacteria may harbor ARGs that affect human health. In the present study, the genotypic analysis of camel milk demonstrated the presence of ARGs that mediate resistance to 18 classes of antibiotics, with resistance to fluoroquinolones and disinfecting agents being the highest. The main resistance mechanisms identified were antibiotic efflux and antibiotic target alteration. These results are in accordance with a recent study on the antibiotic resistome of Bactrian camel milk, where the ARGs with resistance to β-lactam and fluoroquinolone were among the most prevalent resistant genes and the antibiotic inactivation and efflux pump were the major resistance mechanisms reported [14]. Ten bacterial genera were predicted to host the ARGs, namely, unclassified *Bacilli*, *Streptococcus*, *Enterococcus*, *unclassified Gammaproteobacteria*, *unclassified Bacillota*, *unclassified Pseudomonadota*, *Lactococcus*, *Klebsiella*, *Acinetobacter*, and *Paenibacillus*. Consistent with our results, recent studies have highlighted that raw milk acts as a reservoir for ARGs and contributes to their transmission and dissemination. Moreover, the genera *Acinetobacter*, *Klebsiella*, *Lactococcus*, *Staphylococcus*, and *Enterococcus* were predicted to be among the genera harboring AMR genes in bulk tank bovine milk filters [3]. The prediction of genera hosting AMR genes in this study was further confirmed using a culture-based approach, which allowed the identification of these resistant genera, among others.

Resistance may be acquired and spread by horizontal gene transfer involving mobile genetic elements (MGE) including plasmids, insertion sequences, and transposons. Acquiring resistance genes is an important resistance mechanism that mediates the widespread distribution of antibiotic resistance [27]. Plasmids are important genetic carriers of AMR genes with significant clinical implications due to their ability to mobilize or conjugate [23]. MGE promote intracellular and intercellular DNA mobility [28]. Our results demonstrated the possible transmission of the ARGs to other bacteria through MGE. Genes of resistance to several antibiotic classes were predicted to be carried on plasmids and were surrounded by MGE, and some of these genes were part of insertion sequences and transposons. The data presented here provide important insights into the role of camel milk in the spread of AMR.

In vitro antimicrobial susceptibility testing was employed to determine the phenotypic resistance profile of the bacteria isolated from camel milk. The results indicated that 80% of the isolates were resistant to different classes of antibiotics, including multidrug-resistant pathogens genetically identified as *Klebsiella pneumoniae*, *Escherichia coli*, and *Enterobacter hormaechei*. The highest resistance was observed against three antibiotic classes: penicillin, tetracyclines, and carbapenems. These results indicate that these classes of antibiotics are widely used in camel farms. These findings are in accordance with the genotypic results, where penam and tetracycline were among the most frequently detected AMR classes across the majority of the samples, and carbapenem was among the next most prominent AMR classes. Moreover, the genotypic prediction of genera hosting AMR genes was further confirmed by the culture-based approach, which allowed the identification of the resistant genera *Enterococcus*, *Acinetobacter*, and other resistant genera belonging to the *Gammaproteobacteria* class, including *Klebsiella*, *Escherichia*, *Enterobacter*, and *Citrobacter*. In line with the results of this study, the resistance of *Klebsiella pneumonia* and *Escherichia coli* isolates from raw and fermented camel milk in Kenya and Somalia to ampicillin and ceftazidime was reported [29]. Furthermore, *Klebsiella* and *Escherichia coli* were linked in previous studies to spoilage and food safety issues [30].

These findings emphasize the human health risks related to the presence of pathogenic-resistant bacteria, including multi-drug-resistant bacteria. In addition, the possible transmission of the ARGs to other pathogens should be regarded as a public health concern. This highlights the necessity of raising recommendations to implement good veterinary practice and prudent use of guidelines according to WHO [31] in addition to improving the hygienic and storage practices of farms and retail stores, as good-quality raw milk can be successfully stored at 4 °C for up to 4 days with little effect on its microbiological quality [32]. Consumers need to be advised about the safety risks associated with consuming unprocessed milk.

## 4. Material and Methods

### 4.1. Sample Collection and Genomic DNA Extraction

Eight pooled raw dromedary camel milk samples, representative of multiple camels distributed in the Kuwait Desert, were collected from camel milk retail stores. These stores collect fresh milk daily from a large number of camels belonging to camel owners in the Kuwait Desert. The samples were transported in an ice box to the laboratory for genomic DNA extraction. DNA from the collected milk samples was extracted as follows: 2 mL of milk was mixed thoroughly and centrifuged at 10,000× *g* for 10 min, then the fat layer was discarded, and the pellet was subjected to a pre-extraction step using the commercial MolYsis Basic5 kit (Molzym, Bermen, Germany). Then, the genomic DNA was extracted using the GenElute Bacterial Genomic DNA Kit (Sigma-Aldrich, St. Louis, MO, USA) according to the manufacturer’s instructions. DNA concentration was measured using a Qubit 3.0 Fluorometer with the Qubit dsDNA HS Assay Kit (Thermo Fisher Scientific, Waltham, MA, USA). DNA integrity was evaluated using agarose gel electrophoresis. The qualified samples were submitted for library preparation.

### 4.2. Library Preparation and Sequencing

Short insert libraries were prepared. Genomic DNA was sheared by sonication and the purification of DNA fragments with an average size of 200–400 bp was performed using Agencourt Ampure XP beads (Beckman Coulter, Indianapolis, IN, USA). End-repair and 3′-adenylation of the fragments were carried out. Adapters were ligated to the ends of 3′-adenylated fragments. PCR amplification was executed for the fragments with adapters, followed by purification using Agencourt AMPure XP beads. Then, heat-denaturation of the double-stranded PCR products was performed, and they were circularized by the splint oligo sequence forming single-stranded circular DNA. Through rolling-cycle replication, single-stranded circular DNA molecules formed DNA nanoballs (DNBs) consisting of 300+ copies. DNBs were loaded into patterned nanoarrays using a high-intensity DNA nanochip technique. Then, they were sequenced using combinatorial Probe-Anchor Synthesis on an MGI DNBSEQ-G400 in 2 × 150 bp mode.

### 4.3. Bioinformatics Analysis

Read quality check and contaminant removal: Raw sequencing data were demultiplexed in the course of the sequencing process. The quality of the demultiplexed reads was checked using FastQC v0.11.7 [33]. Undesired sequences were removed from raw reads using SNAP v2.0.2 [34]. The CamDro3 genome was used as a host reference.

Read quality trimming, recalibration, and error correction: Read quality trimming was performed using the BBTools package v39.01 [35]. This comprised the removal of duplicate reads, adapter sequences, low-entropy reads, and trimming of bases with quality scores < 20. Reads with invalid or ambiguous bases and reads with a length <127 base pairs were discarded. Only reads passing the quality trimming as pairs entered downstream analysis. Read quality recalibration and error correction were performed using the BBTools package v39.01 [35]. Quality-trimmed reads were aligned to a preliminary de-novo assembly made using Tadpole from a subset of the quality-trimmed reads. Alignment information was used to recalibrate the base quality of all quality-trimmed reads. Sequencing errors were corrected by consecutively applying the BBTools programs BBMerge, Clumpify, and Tadpole in error-correction mode on the quality-recalibrated reads.

Normalization, de-novo assembly, self-referenced mapping, and assembly quality filtering: 31-bp kmers of filtered reads were normalized using BBNorm to a target kmer depth of 100×, with a minimum kmer depth of 3×. A cross-assembly of these normalized reads from all samples was constructed with MEGAHIT v1.1.4–2-gd1998a1 [36,37]. The program was run with the preset “meta-large” and a minimum contig length of 1000 bp. Scaffolds produced by MEGAHIT were subject to an additional scaffolding step by SSPACE standard v3.0 [38] with default parameters.

Filtered reads were back-mapped onto the primary assembly using BBMap from the BBTools package v39.01 [35]. For each contig in the primary assembly, the overall coverage was determined from the number of unambiguously mapped reads. Contigs of length ≥ 1000 bp and coverage ≥ 10× were considered reliable and are referred to as “filtered assembly”. Statistics on the primary and filtered assembly were produced using code from the “Assemblathon 2” project [39].

Read counts: Per-sample read counts were produced using SAMtools v1.9 [40] by mapping filtered reads to filtered contigs produced in the self-referenced mapping step. A MAPQ alignment quality score of ≥30 was required for a read to map. The numbers of read counts remaining after the selected processing step are summarized in Table S1. The summary was generated in v4.3.1 [41] using the R package data.table v1.14.8 [42] and ggplot2 v3.4.4 [43].

Ribosomal RNA gene detection, gene calling, and annotation: Ribosomal RNA (rRNA) genes were detected using cmscan v1.1.4 [44] with the Rfam database from 2022-11-10 [45]. Hits were pre-filtered for an E-value ≤ 10^−6^. Gene sequences (“coding sequences”, CDS) were identified using Prodigal v2.6.3 [46] in “meta” mode using genetic code 11. A subset of CDS with length ≥ 80 amino acids was generated for subsequent annotation, henceforward referred to as “filtered CDS”.

Two classification tools were used to annotate the CDS obtained in gene calling: First, all CDS were subject to accelerated BLASTP annotation [47] by DIAMOND v2.0.8 [48] against NCBI non-redundant protein sequences [49]. Hits were pre-filtered for an E-value ≤ 10^−6^, retaining top-scoring hits. Second, filtered CDS were classified by InterProScan v5.64-96.0 [50] according to Pfam [51]. Hits were pre-filtered for an E-value ≤ 10^−6^. For overlapping domains, the highest-scoring hit was retained.

In addition to the gene annotation described above, the following annotations were performed:

All CDS: Resistance genes by CARD Resistance Gene Identifier [21], “perfect” and “strict” hits and mobile elements with ISfinder [52], E-value ≤ 10^−6^.

Filtered contigs: Plasmid identification by Platon v1.6 [23,53,54,55], E-value ≤ 10^−6^.

Genome binning and taxonomic assignment: Contigs were assigned to genome bins (aka. Metagenome-Assembled Genomes, MAGs) using MetaBAT v2.12.1 [56] with a minimum contig size of 2500 bp and a minimum bin size (cumulative contig length) of 50,000 bp.

Taxonomic assignment of contigs and genome bins was accomplished with the Contig Annotation Tool (CAT) and Bin Annotation Tool (BAT), respectively, v5.2.3, based on database matches inferred by DIAMOND during gene annotation. Taxonomic information was taken from the pre-compiled CAT taxonomy database of 2021-01-07. Hierarchical taxonomic classifications of contigs were in compliance with NCBI taxonomy [57] at standard taxonomic levels.

### 4.4. Bacterial Isolation and Identification

Upon arrival at the laboratory, collected camel milk samples were subjected to bacterial isolation on selective media for pathogens according to Gao et al. [58]. The collected samples were enriched as follows: ten milliliters of enrichment media were inoculated with 1 mL of collected milk and incubated at 37 °C overnight. After the enrichment process, 10 μL of each enriched sample was spread onto general media (nutrient agar) and selective media (MacConkey agar) plates (Thermo Fisher Scientific, Waltham, MA, USA) and incubated at 37 °C for 24 h. After purification, genomic DNA extraction from overnight cultures of pure bacterial colonies was performed using the GenElute™ Bacterial Genomic DNA Kit (Sigma-Aldrich, USA). The quantity and purity of the extracted DNA was determined as mentioned above. The 16S rRNA genes were amplified using universal primers 27F (5′-AGAGTTTGATCCTGGCTCAG-3′) and U1492R (5′-CTACGGCTACCTTGTTACGA-3′) and sequenced using the Applied Biosystems 3730 Genetic Analyzer.

### 4.5. Resistance Profile of Bacteria

The in vitro antibiogram profile of the genetically identified isolates (*Klebsiella pneumoniae*, *Escherichia coli*, *Acinetobacter* sp., *Enterobacter* sp., *Citrobacter* sp., and *Enterococcus durans*) was determined using the disk diffusion method following the Clinical Laboratory Standards Institute CLSI guidelines [25]. The susceptibility test included a panel of selected antibiotics that the dairy farmers commonly use in the treatment of infections in Kuwait in addition to other antibiotics. The list of the antibiotics used in this study includes ampicillin (10 μg/mL), amoxicillin/clavulanate (30 μg/mL), tetracycline (30 μg/mL), doxycycline (30 μg/mL), erythromycin (15 μg/mL), chloramphenicol (30 μg/mL), ceftazidime (30 μg/mL), meropenem (10 μg/mL), imipenem (10 μg/mL), Kanamycin (30 μg/mL), streptomycin (10 μg/mL), and ciprofloxacin (5 μg/mL) (Oxoid^TM^, Thermo Fisher Scientific, USA). The CLSI routine quality control recommendations were followed, and the resistance was defined according to the clinical and laboratory standards institute [25].

## 5. Conclusions

In this study, the application of two approaches, shotgun sequencing and in vitro antimicrobial susceptibility testing, allowed characterization of the antibiotic-resistant profile of bacteria and their AMR genes in dromedary camel milk. This study clearly demonstrated that retail raw camel milk is a source of ARGs mediating resistance to 18 classes of antibiotics, disinfecting agents, and antiseptics. Furthermore, this study pointed out the possible transmission of the resistance genes to other pathogenic bacteria mediated by the mobile genetic elements carrying AMR genes. In addition, the identification of MDR *Enterobacterales*, particularly *Klebsiella pneumonia* and *Escherichia coli*, which are linked to spoilage and food safety issues, is considered a serious public health problem. These findings highlight the food safety risks to human health associated with the handling and consumption of raw camel milk and emphasize the need to enhance hygienic practices in farms and retail stores and ensure proper transportation and storage to limit the prevalence of AMR genes in milk and protect consumers. Further investigations evaluating a large number of samples using long-read shotgun metagenomics sequencing that generates long sequencing reads are necessary to deeply understand the resistome of camel milk.

## Figures and Tables

**Table 1:** Group means and standard deviations of relative abundances of drug classes.

Drug Class	Mean	SD
fluoroquinolone antibiotic	12.48	1.68
disinfecting agents and antiseptics	9.02	1.85
cephalosporin	8.42	1.26
penam	8.07	1.23
tetracycline antibiotic	8.06	1.20
peptide antibiotic	5.92	1.84
glycopeptide antibiotic	5.62	5.21
rifamycin antibiotic	5.29	0.84
aminoglycoside antibiotic	5.27	2.34
macrolide antibiotic	4.84	1.01
phenicol antibiotic	4.25	0.59
cephamycin	3.87	1.18
glycylcycline	3.83	0.67
carbapenem	3.35	0.76
penem	3.21	0.60
monobactam	2.01	0.55
phosphonic acid antibiotic	1.92	0.52
aminocoumarin antibiotic	1.18	0.24
nucleoside antibiotic	1.13	0.27
diaminopyrimidine antibiotic	0.84	0.17
nitroimidazole antibiotic	0.47	0.19
lincosamide antibiotic	0.29	0.47
nitrofuran antibiotic	0.23	0.29
streptogramin antibiotic	0.14	0.22
sulfonamide antibiotic	0.12	0.05
pleuromutilin antibiotic	0.10	0.17
streptogramin A antibiotic	0.03	0.05
streptogramin B antibiotic	0.03	0.05

**Table 2:** Relative abundances of genes of resistance, drug classes, resistance mechanisms, AMR gene families, and taxonomic rank (genus), as identified using CARD across samples.

Genes of Resistance	Drug Class	Resistance Mechanism	AMR Gene Family	Genus	CM 007	CM 009	CM 011	CM 013	CM 014	CM 015	CM 027	CM 028
vanY gene in vanB cluster	glycopeptide antibiotic	antibiotic target alteration	vanY	*Enterococcus*	0.29	1.53	0.08	0.09	0.09	0.15	0.09	0.22
vanT gene in vanG cluster	glycopeptide antibiotic	antibiotic target alteration	vanT	*Enterococcus*	0.18	0.98	0.05	0.05	0.04	0.12	0.05	0.15
vanW gene in vanG cluster	glycopeptide antibiotic	antibiotic target alteration	vanW	*Streptococcus*	0.42	0.20	0.00	0.00	0.03	0.02	0.01	0.06
patB	fluoroquinolone antibiotic	antibiotic efflux	ATP-binding cassette (ABC) antibiotic efflux pump	*Streptococcus*	0.40	0.18	0.00	0.00	0.00	0.02	0.01	0.06
vanY gene in vanF cluster	glycopeptide antibiotic	antibiotic target alteration	vanY	*Streptococcus*	0.35	0.16	0.00	0.00	0.02	0.02	0.00	0.05
vanY gene in vanM cluster	glycopeptide antibiotic	antibiotic target alteration	vanY	*Streptococcus*	0.35	0.16	0.00	0.00	0.02	0.02	0.00	0.05
qacJ	disinfecting agents and antiseptics	antibiotic efflux	small multidrug resistance (SMR) antibiotic efflux pump	*Streptococcus*	0.14	0.06	0.00	0.00	0.01	0.01	0.00	0.02
mreA	macrolide antibiotic	antibiotic efflux	major facilitator superfamily (MFS) antibiotic efflux pump	*Streptococcus*	0.09	0.05	0.00	0.00	0.02	0.01	0.00	0.02
Streptococcus agalactiae mprF	peptide antibiotic	antibiotic target alteration	defensin resistant mprF	*Streptococcus*	0.00	0.00	0.01	0.01	0.06	0.00	0.00	0.01
lmrD	lincosamide antibiotic	antibiotic efflux	ATP-binding cassette (ABC) antibiotic efflux pump	*Lactococcus*	0.01	0.11	0.00	0.00	0.00	0.00	0.00	0.02
vanY gene in vanG cluster	glycopeptide antibiotic	antibiotic target alteration	vanY	*Lactococcus*	0.02	0.08	0.00	0.00	0.00	0.00	0.00	0.01
CrcB	aminoglycoside antibiotic	antibiotic efflux	multidrug and toxic compound extrusion (MATE) transporter	*Klebsiella*	0.00	0.00	0.00	0.00	0.00	0.06	0.00	0.00
adeC	glycylcycline	antibiotic efflux	resistance-nodulation-cell division (RND) antibiotic efflux pump	*Acinetobacter*	0.00	0.00	0.01	0.01	0.01	0.00	0.01	0.01
adeC	tetracycline antibiotic	antibiotic efflux	resistance-nodulation-cell division (RND) antibiotic efflux pump	*Acinetobacter*	0.00	0.00	0.01	0.01	0.01	0.00	0.01	0.01
vanY gene in vanB cluster	glycopeptide antibiotic	antibiotic target alteration	vanY	*Paenibacillus*	0.00	0.00	0.00	0.00	0.02	0.00	0.00	0.00
tet(36)	tetracycline antibiotic	antibiotic target protection	tetracycline-resistant ribosomal protection protein	*Paenibacillus*	0.00	0.00	0.00	0.00	0.01	0.00	0.00	0.00
vanW gene in vanI cluster	glycopeptide antibiotic	antibiotic target alteration	vanW	*Paenibacillus*	0.00	0.00	0.00	0.00	0.01	0.00	0.00	0.00

**Table 3:** Resistance genes within a 10-CDS distance of mobile elements, as identified by ISFinder.

Contig	On Plasmid	CDS ID	Target Distance	ISfinder Description	CARD Description	CARD Drug Class	CARD Resistance Mechanism
1450	1	1450_5	7, −1, 1		lnuB	lincosamide	antibiotic inactivation
1450	1	1450_6	6, −2, 0	ISRe46_aa4	lsaE	lincosamide; streptogramin; pleuromutilin,	antibiotic target protection
1450	1	1450_6	6, −2, 0	ISRe46_aa4	lsaE	lincosamide; streptogramin; pleuromutilin,	antibiotic target protection
2967	1	2967_3	−2, −1		tet(A)	tetracycline	antibiotic efflux
3207	1	3207_1	4		EreA	macrolide	antibiotic inactivation
3207	1	3207_2	3		qacEdelta1	disinfecting agents and antiseptics	antibiotic efflux
3207	1	3207_3	2		sul1	sulfonamide	antibiotic target replacement
3238	1	3238_1	0, 1	Tn2_aa1	TEM-1	monobactam; cephalosporin; penam; penem	antibiotic inactivation
3348	1	3348_1	1, 5		qacL	disinfecting agents and antiseptics	antibiotic efflux
3348	1	3348_3	−1, 3		sul3	sulfonamide	antibiotic target replacement
3369	1	3369_1	0	ISArsp14_aa11	tet(45)	tetracycline	antibiotic efflux
4993	1	4993_2	0, 1	Tn2_aa1	CTX-M-15	cephalosporin; penam	antibiotic inactivation
6435	1	6435_1	0	MICBce1_aa1	DHA-1	cephalosporin; cephamycin	antibiotic inactivation
7063	1	7063_1	0	Tn2_aa1	LAP-2	cephalosporin; penam; penem	antibiotic inactivation
9281	1	9281_1	0	ISAba61_aa1	cmlA1	phenicol	antibiotic efflux
10144	1	10144_2	−1		QnrS1	fluoroquinolone	antibiotic target protection
13504	1	13504_1	0	ISBce8_aa2	AAC(6′)-Ib-cr6	fluoroquinolone; aminoglycoside	antibiotic inactivation
13534	1	13534_1	0	ISCco2_aa4	APH(3′)-IIIa	aminoglycoside	antibiotic inactivation
14655	1	14655_1	0	ISSsu9_aa2	dfrA12	diaminopyrimidine	antibiotic target replacement
16142	1	16142_1	0	ISRe46_aa6	ErmB	macrolide; lincosamid; streptogramin; streptogramin A; streptogramin B	antibiotic target alteration

**Table 4:** Resistance genes within a 10-CDS distance of mobile elements associated with bacterial genera.

Genus	Contig	On Plasmid	CDS ID	Target Distance	ISfinder Description	CARD Description	CARD Drug Class	CARD Resistance Mechanism
*Paenibacillus*	1	0	1_943	4		vanT gene in vanG cluster	glycopeptide antibiotic	antibiotic target alteration
*Paenibacillus*	3	0	3_46	−1		vanW gene in vanI cluster	glycopeptide antibiotic	antibiotic target alteration
*Paenibacillus*	3	0	3_546	−8, 7, 8, 9		tet(36)	tetracycline antibiotic	antibiotic target protection
*Paenibacillus*	4	0	4_279	−5, 4, 9, 10		vanY gene in vanB cluster	glycopeptide antibiotic	antibiotic target alteration
*Paenibacillus*	9	0	9_284	−8, −2, 4		LlmA 23S ribosomal RNA methyltransferase	lincosamide antibiotic	antibiotic target alteration
*Paenibacillus*	9	0	9_367	0, 1, 9	ISArsp14_aa11	tet(45)	tetracycline antibiotic	antibiotic efflux
*Paenibacillus*	20	0	20_197	8		vanH gene in vanO cluster	glycopeptide antibiotic	antibiotic target alteration
*Enterococcus*	39	0	39_47	1		vanY gene in vanB cluster	glycopeptide antibiotic	antibiotic target alteration
*Enterococcus*	111	0	111_30	−6		vanT gene in vanG cluster	glycopeptide antibiotic	antibiotic target alteration
*Enterococcus*	111	0	111_30	−6		vanT gene in vanG cluster	glycopeptide antibiotic	antibiotic target alteration
*Streptococcus*	228	0	228_29	7		vanY gene in vanM cluster	glycopeptide antibiotic	antibiotic target alteration
*Streptococcus*	242	0	242_12	0, 1	ISArsp14_aa5	patB	fluoroquinolone antibiotic	antibiotic efflux
*Streptococcus*	679	0	679_11	−2		Streptococcus agalactiae mprF	peptide antibiotic	antibiotic target alteration
*Streptococcus*	758	0	758_6	7, 8		mreA	macrolide antibiotic	antibiotic efflux
*Streptococcus*	854	0	854_16	−6, −5, −8		qacJ	disinfecting agents and antiseptics	antibiotic efflux
*Lactococcus*	1373	0	1373_2	−1, 9, 0	ISArsp14_aa5	lmrD	lincosamide antibiotic	antibiotic efflux

**Table 5:** Antibiotic resistance profile of *Enterobacterales* isolates from camel milk.

Antibiotic	Antibiotic Class	Disk Content	Interpretive Categories and Zone Diameter Breakpoints (Nearest Whole mm)			Isolates																	
						*Klebsiella pneumonia*						*Escherichia coli*						*Enterobacter* sp.					
						No. of Isolates			(%)			No. of Isolates			(%)			No. of Isolates			(%)		
			S	I	R	S	I	R	S	I	R	S	I	R	S	I	R	S	I	R	S	I	R
Ampicillin	Penicillin	10 µg	≥17	14–16	≤13	0	0	12	0	0	100	3	0	2	60	0	40	3	0	2	20	60	20
AMC	Penicillin	30 µg	≥18	14–17	≤13	11	1	0	92	8	0	5	0	0	100	0	0	5	0	0	20	0	80
Ceftazidime	Cephems	30 µg	≥21	18–20	≤17	8	2	2	67	17	17	5	0	0	100	0	0	5	0	0	100	0	0
Meropenem	Carbapenems	10 µg	≥23	20–22	≤19	0	0	12	0	0	100	5	0	0	100	0	0	5	0	0	100	0	0
Imipenem	Carbapenems	10 µg	≥23	20–22	≤19	12	0	0	100	0	0	4	1	0	80	20	0	4	1	0	100	0	0
Gentamicin	Aminoglycosides	10 µg	≥18	15–17	≤14	11	1	0	92	8	0	5	0	0	100	0	0	5	0	0	100	0	0
Kanamycin	Aminoglycosides	30 µg	≥18	14–17	≤13	8	0	4	67	0	33	5	0	0	100	0	0	5	0	0	100	0	0
Streptomycin	Aminoglycosides	10 µg	≥15	12–14	≤11	-	-	-	-	-	-	2	0	3	40	0	60	2	0	3	60	0	40
Tetracycline	Tetracyclines	30 µg	≥15	12–14	≤11	5	0	7	42	0	58	2	0	3	40	0	60	2	0	3	60	20	20
Doxycycline	Tetracyclines	30 µg	≥14	11–13	≤10	6	0	6	50	0	50	3	0	2	60	0	40	3	0	2	60	20	20
Ciprofloxacin	Fluoroquinolones	5 µg	≥26	22–25	≤21	2	7	3	17	58	25	3	1	1	60	20	20	3	1	1	20	60	20
Chloramphenicol	Phenicols	30 µg	≥18	13–17	≤12	11	0	1	92	0	8	5	0	0	100	0	0	5	0	0	100	0	0

AMC: Amoxicillin/clavulanate; -: Not Tested. S: Susceptible; I: Intermediate; R: Resistant.

## Data Availability

The high-throughput sequencing data were deposited in the NCBI database under the BioProject ID: PRJNA1070879.

## Supplementary Materials

The following supporting information can be downloaded at: https://www.mdpi.com/article/10.3390/antibiotics13050380/s1, Table S1: The numbers of read counts remaining after selected processing step.
