# Supplementary material for: Camel Milk Resistome in Kuwait: Genotypic and Phenotypic Characterization
Source: Antibiotics (Basel). 2024 Apr 23;13(5):380. doi: 10.3390/antibiotics13050380 (PMC11117293; doi:10.3390/antibiotics13050380)
Supplement: Supplementary file 1 [file antibiotics-13-00380-s001.zip › antibiotics-2962547-supplementary.pdf]

Camel milk resistome: genotypic and phenotypic characterization

Table S1. The numbers of read counts remaining after selected processing step

Sample ID	Raw	Decontaminated	Filtered	Mapped to Contigs
CM_007	48585364	27733006	21397996	2838883
CM_009	48641326	26610272	21072204	17078334
CM_011	48282716	23624684	17820172	12797469
CM_013	48434292	23807964	17899420	12914118
CM_014	48668986	16587446	12635028	2134738
CM_015	48604458	46440846	36890372	3909521
CM_027	48334188	22305436	16549512	9191950
CM_028	48563774	27400450	21073080	15303608
